# Mass Spectrometric Measurements of 11‐Deoxycortisol, Androstenedione and Dehydroepiandrosterone Are Superior to Cortisol to Assess Selectivity of Non‐Stimulated Adrenal Vein Sampling

**DOI:** 10.1111/cen.70037

**Published:** 2025-09-16

**Authors:** Francesco Alessi, Christina Pamporaki, Mirko Peitzsch, Georgiana Constantinescu, Hanna Remde, Lydia Kürzinger, Carmina T. Fuss, Manuel Schulze, Sybille Fuld, Sradha Kotwal, Jun Yang, Martin Reincke, Felix Beuschlein, Jacques W. M. Lenders, Graeme Eisenhofer

**Affiliations:** ^1^ Department of Medicine III University Hospital Carl Gustav Carus, Technische Universität Dresden Dresden Germany; ^2^ Institute of Clinical Chemistry and Laboratory Medicine Medical Faculty and University Hospital Carl Gustav Carus, Technische Universität Dresden Dresden Germany; ^3^ Department of Internal Medicine I, Division of Endocrinology and Diabetes University Hospital, University of Würzburg Würzburg Germany; ^4^ Department Information Services and High Performance Computing, Center for Interdisciplinary Digital Sciences Technische Universität Dresden Dresden Germany; ^5^ Department of Nephrology, Prince of Wales Hospital, Renal and Metabolic Division, The George Institute for Global Health UNSW Sydney Sydney New South Wales Australia; ^6^ Centre for Endocrinology and Metabolism Hudson Institute of Medical Research Clayton Victoria Australia; ^7^ Department of Medicine IV University Hospital, Ludwig Maximilian University Munich Munich Germany; ^8^ Department of Endocrinology, Diabetology and Clinical Nutrition University Hospital Zurich and the LOOP Zurich Medical Research Center Zurich Switzerland; ^9^ Department of Internal Medicine Radboud University Medical Center Nijmegen the Netherlands

**Keywords:** 11‐deoxycortisol, adrenal venous sampling, androstenedione, cortisol, dehydroepiandrosterone, primary aldosteronism, selectivity index

## Abstract

**Objective:**

Successful adrenal venous sampling (AVS) is traditionally assessed using the ratio of cortisol in adrenal to peripheral venous plasma to calculate the selectivity index. With mass spectrometry other steroids can be simultaneously measured that may improve numbers of apparent successful sampling procedures.

**Design:**

Cross‐sectional multicenter study.

**Patients:**

The study involved 229 patients who underwent unstimulated AVS for subtyping primary aldosteronism.

**Measurements:**

Adrenal and peripheral venous plasma cortisol, 11‐deoxycortisol, androstenedione and dehydroepiandrosterone were measured by mass spectrometry to assess AVS selectivity. Ratios of aldosterone, measured by mass spectrometry, to each of the four steroids in right versus left adrenal venous plasma were used to assess lateralisation.

**Results:**

Selectivity indices for 11‐deoxycortisol, androstenedione and dehydroepiandrosterone were respectively 5.7–(CI 5.1–6.2), 5.1–(CI 4.8–5.4) and 5.9–(CI 5.5–6.4) fold higher (*p* < 0.0001) than for cortisol. At selectivity index cut‐offs of ≥ 3 and ≥ 2, rates of bilateral successful AVS were respectively 91% and 93% for 11‐deoxycortisol, 90% and 91% for androstenedione and 89% and 91% for dehydroepiandrosterone, all higher (*p* < 0.0001) than the 69% and 85% respective success rates for cortisol. With 11‐deoxycortisol, rates of apparent unsuccessful AVS procedures were reduced by 54%–72% compared to use of cortisol. There were no clear advantages of any single steroid over the others to assess lateralized aldosterone secretion.

**Conclusions:**

11‐Deoxycortisol, androstenedione and dehydroepiandrosterone are more suitable biomarkers than cortisol to assess AVS selectivity and allow rescue of many procedures otherwise deemed unsuccessful. Measurements of cortisol to assess AVS selectivity are best replaced by mass spectrometric measurements of other steroids.

## 1 Introduction

Primary aldosteronism (PA), characterised by inappropriately high aldosterone secretion that eludes physiological feedback control, is a common but underdiagnosed cause of secondary hypertension [1]. Diagnosis requires a multistep approach that culminates in adrenal venous sampling (AVS), recommended to distinguish unilateral from bilateral adrenal over‐secretion of aldosterone [2, 3]. This final step is crucial since unilateral PA is curable by adrenalectomy, whereas bilateral PA is best treated with mineralocorticoid receptor antagonists. AVS is a technically demanding procedure for which successful catheterisation of both adrenal veins is often difficult. Principal factors linked to unsuccessful catheterisation are limited experience of the operator and anatomic variations of the adrenal veins [4, 5, 6]. The major challenge operators face is the catheterisation of the right adrenal vein, which is narrow, short, and drains into the inferior vena cava at a perpendicular angle [5]. Anatomical variations of adrenal veins, such as drainage into a common trunk of the accessory hepatic vein or into the inferior phrenic vein (usually left), and supernumerary small adrenal veins can further complicate AVS even in experienced hands [6, 7].

Correct positioning of catheters is verified from the ratio of cortisol in an adrenal vein versus a peripheral vein, usually the inferior vena cava [8]. The resulting ratio is termed the selectivity index. Arbitrarily defined ratios of ≥ 2 or ≥ 3 are commonly employed to confirm successful catheterisation, the former usually employed for procedures without cosyntropin stimulation and the latter with stimulation [8]. Based on these cut‐offs, many AVS studies are considered non‐selective, with usual failure rates of 20% [9] but reaching 50% [10]. Nonselective AVS usually fails to provide sufficient information for subtyping, necessitating repeated procedures that increase financial costs, burdens on time and procedure‐related risks.

The use of cortisol to ascertain success of AVS has several drawbacks. Stress associated variations in circulating cortisol and co‐secretion of cortisol with aldosterone are two factors that may compromise assessments of AVS selectivity [11]. The major downside, however, is the long circulatory half‐life of cortisol (> 60 min), which reflects its slow clearance further complicated by impacts of cortisol‐binding globulin [12, 13]. Consequently, peripheral venous to adrenal venous gradients of plasma cortisol are much lower than those of adrenal biomarkers with rapid circulatory clearances. Such biomarkers include metanephrine, which has a plasma half‐life of a few minutes and is produced from epinephrine within adrenal chromaffin cells by a process independent of epinephrine secretion [14]. Hence, this metabolite shows a sixfold higher peripheral to adrenal venous plasma concentration gradient than cortisol and is superior to cortisol for assessment of AVS selectivity [15, 16].

Stimulation with cosyntropin overcomes some of the aforementioned issues by enhancing cortisol secretion to improve assessment of AVS selectivity [17, 18]. However, this increases the complexity of an already demanding procedure, adds to costs and appears to underestimate unilateral PA compared to unstimulated sampling [19, 20]. Furthermore, findings of a randomised controlled trial indicate that cosyntropin‐stimulated sampling offers no benefit for post‐adrenalectomy clinical outcomes compared to unstimulated sampling [21].

With the above background in mind, there is clear need for alternative biomarkers to cortisol to assess selectivity of AVS, particularly for unstimulated sampling. Since mass spectrometry offers an advantage to simultaneously measure multiple steroids in a single sample, we focused on a panel of steroids as biomarkers rather than separate measurement of metanephrine. Specifically, given our previous findings that plasma 11‐deoxycortisol, androstenedione and dehydroepiandrosterone (DHEA) show larger peripheral to adrenal venous gradients than cortisol [22], we hypothesised that those steroids would improve confirmation of successful AVS. The objective of this study was therefore to investigate whether use of those steroids during unstimulated AVS might rescue some procedures that would otherwise be classified as nonselective based on cortisol.

## 2 Materials and Methods

### 2.1 Patients

Patients were enroled into the PROspective study on the diagnostic value of the Steroid profiling in primary ALDOsteronism (PROSALDO, registration no: DRKS00017084) at 7 tertiary care centres: University Hospitals Dresden, Würzburg and Munich in Germany; University Hospital Zurich in Switzerland; Hudson Institute of Medical Research, Clayton, Victoria and Prince of Wales and St George Hospitals, Sydney, Australia. Entry into the protocol required suspicion of PA based on hypertension and at least one of several other criteria: (1) office blood pressure above 150/100 mmHg on two separate visits; (2) therapy resistant hypertension with at least three different antihypertensives, including one diuretic; (3) spontaneous or diuretic‐induced hypokalemia; (4) an adrenal incidentaloma; (5) family history of PA, early onset hypertension or cerebrovascular accident (< 40 years age); or (6) hypertension and obstructive sleep apnea.

Patients were excluded from the protocol based on several criteria: (1) presence of other forms of secondary hypertension; (2) necessity for continued use of medications that interfere with laboratory test results; (3) established low plasma concentrations of aldosterone (< 170 pmol/L by immunoassay measurements); (4) pregnancy; (5) impaired mental capacity that precludes informed consent; (6) glucocorticoid remedial hyperaldosteronism; and (7) severe or terminal co‐morbidity that would preclude required investigational procedures and/or therapeutic interventions.

According to consideration of the above inclusion and exclusion criteria, there were 978 patients (502 females, 476 males, median age 50) enroled into PROSALDO between January 2019 and April 2025. All patients provided written informed consent under the protocol, which was approved by the ethics committees at each study centre.

### 2.2 Clinical Test Procedures

**FIGURE 1:**
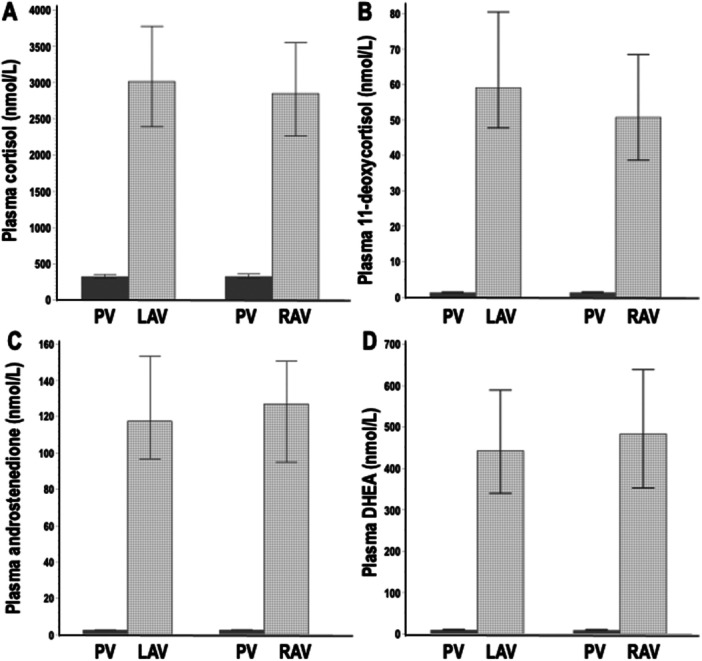
Plasma concentrations of cortisol (A), 11‐deoxycortisol (B), androstenedione (C) and DHEA (D) from blood samples derived from peripheral veins (PV), left adrenal veins (LAV) and right adrenal veins (RAV). Data are shown as geometric means with 95% confidence intervals.

The design features of the PROSALDO trial have been documented previously [23, 24, 25], and are further detailed in the Supporting Information S1: Figure 1 and the associated text of the supplemental appendix. In brief, the protocol required patients to first undergo screening for PA according to measurements of plasma aldosterone, renin and potassium performed on two separate occasions with blood samples collected in the morning and seated position. The same samples were also used for steroid profiling by mass spectrometry. Patients underwent a seated saline infusion test if screening returned positive test results for either or both the aldosterone:renin ratio or steroid profiles.

Computed tomography and AVS were performed in patients with positive confirmatory test results, though occasional patients progressed directly to AVS studies based on strong biochemical evidence of PA combined with results of imaging evidence that indicated an adenoma ( > 0.8 mm). AVS was performed under fluoroscopic guidance at all centres without cosyntropin stimulation. Samples were obtained simultaneously from each adrenal vein and the inferior vena cava at five centres, whereas at two centres sampling was sequential and included matching adrenal and peripheral vein samples. As further detailed in the supplemental appendix, outcome assessments in patients who underwent adrenalectomy were undertaken to document biochemical and/or clinical cure.

### 2.3 Mass Spectrometry Measurements

Mass spectrometry‐based measurements of steroids in plasma specimens (EDTA) sent to a central laboratory from each centre were according to a previously established method [22]. Among the steroids measured, those critical to the present analyses included cortisol, 11‐deoxycortisol, androstenedione, DHEA and aldosterone. Inter‐assay coefficients of variations at low/normal, mid and high plasma concentration ranges varied from 7.1% to 11.3% for cortisol, 6.2% to 8.7% for 11‐deoxycortisol, 7.9% to 13.8% for androstenedione, 13.6% to 24.2% for DHEA and 6.9% to 13.1% for aldosterone.

### 2.4 Statistical Analyses

The JMP Pro statistics software package version 18 (SAS Institute Inc., Cary, NC) was used for data analysis. Continuous parameters are expressed as means and 95% confidence intervals (CIs) or if not normally distributed, as medians or geometric means with CIs. Selectivity indices derived from the different steroids, rates of nonselective versus selective samplings and differences in lateralisation indices were compared using the Wilcoxon signed‐rank test for paired data. Receiver‐operating characteristic (ROC) curves and ROC tables were generated to determine optimal cut‐offs for selectivity indices for each of the four examined steroids. For this analysis classification of selective sampling was based on findings of selectivity indices ≥ 2 for three of the four steroids, while anything less was classified as nonselective.

## 3 Results

### 3.1 Patients

From the 978 patients recruited into the PROSALDO trial with suspicion of primary aldosteronism there were 253 who underwent AVS (Supporting Information S1: Figure 1). Among those 253 patients, there were 229 with mass spectrometric measurements of steroids in both adrenal veins who were included in the present cross‐sectional analysis. Twenty patients were excluded because samples from AVS studies were not provided for steroid profiling and four others were excluded because results for steroid profiling were missing from one of the two adrenal veins. The 229 patients in the analysis included 126 males and 103 females with a median age of 50 years (range 21–75).

### 3.2 Adrenal and Peripheral Venous Steroids and Selectivity Indices

Plasma cortisol concentrations in left and right adrenal veins were on average 52‐fold higher than concentrations of 11‐deoxycortisol, 24‐fold higher than concentrations of androstenedione and sixfold higher than for DHEA (Figure 1). Nevertheless, compared to the adrenal veins, differences were larger for peripheral venous plasma where concentrations of cortisol were 257‐fold higher than those of 11‐deoxycortisol, 127‐fold higher than androstenedione and 36‐fold higher than DHEA. Consequently, the relative enrichment of 11‐deoxycortisol, androstenedione and DHEA in adrenal venous versus peripheral venous plasma exceeded that of cortisol.

**FIGURE 2:**
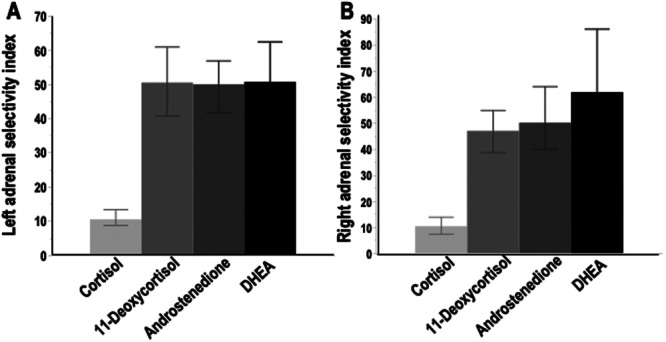
Selectivity indices for cortisol, 11‐deoxycortisol, androstenedione and DHEA for blood samples drawn from left (A) and right (B) adrenal veins.

The aforementioned differences translated into 5.0‐ to 6.1‐fold higher (*p* < 0.0001) selectivity indices for 11‐deoxycortisol, androstenedione and DHEA than those for cortisol (Figure 2). There were no significant differences in selectivity indices between left and right adrenal veins for any of the four steroids. For the combined data of both adrenal veins, selectivity indices for 11‐deoxycortisol, androstenedione and DHEA were respectively 5.7–(CI 5.1–6.2), 5.1–(CI 4.8–5.4) and 5.9–(CI 5.5–6.4) fold higher (*p* < 0.0001) than the selectivity index for cortisol.

### 3.3 AVS Selectivity Scatterplots and Sampling Success

**FIGURE 3:**
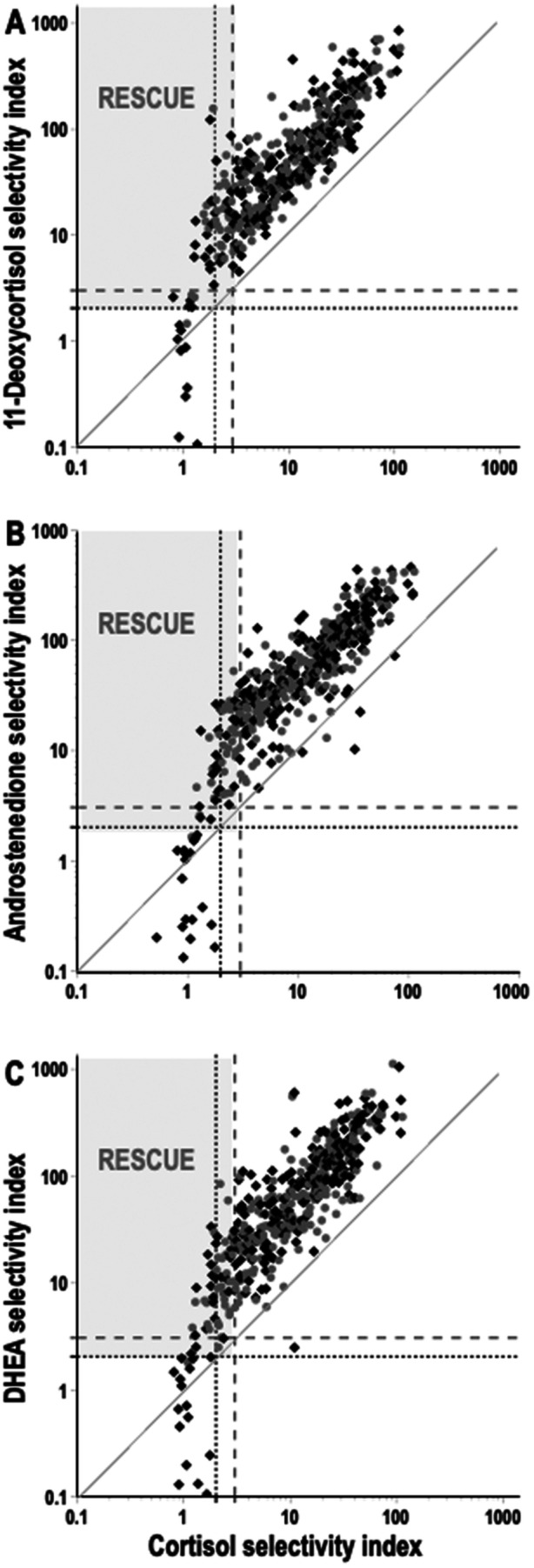
Scatterplot relationships for selectivity indices of cortisol shown in x‐axes versus 11‐deoxycortisol (A), androstenedione (B) and DHEA (C) shown in y‐axes. Note the logarithmic scales for both x‐ and y‐axes. Selectivity index values are shown separately for right (♦) and left (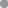) adrenal venous samples. The line of identity is displayed where x = y. Selectivity index cut‐offs for 2 (dotted lines) and 3 (dashed lines) are shown to demarcate selectivity index values below which correct positioning of catheters is indicated to have failed at those cut‐offs. The upper left grey‐area quadrants serve to illustrate samplings that can be considered rescued by use of the alternative steroids to cortisol to assess selectivity.

Scatterplot comparisons of selectivity indices for cortisol as the routine standard (x‐axes) versus those of 11‐deoxycortisol, androstenedione and DHEA (y‐axes) revealed selectivity indices that invariably fell above the line of identity for each of the latter three steroids over the range of selectivity index values from 2 up to a 1000 (Figure 3). For selectivity index cut‐offs of either ≥ 2 or ≥ 3, the shift of relationships to higher selectivity indices for each of the three steroids compared to those of cortisol was associated with a higher (*p* < 0.0001) proportion of failed AVS procedures for cortisol than for the other three steroids.

At a selectivity index cut‐off of ≥ 2 for cortisol, AVS appeared unsuccessful in 33 and eight procedures involving respective right and left adrenal veins, including six procedures that were unsuccessful for both adrenal veins (Figure 3). At the same selectivity index cut‐off of ≥ 2 for 11‐deoxycortisol, AVS appeared unsuccessful in only 15 procedures involving the right adrenal vein, one involving the left adrenal vein and no procedures for both (Figure 3A). All procedures that fell below the cut‐off of ≥ 2 for 11‐deoxycortisol also fell below that cut‐off for cortisol. At the selectivity index cut‐off ≥ 2 for androstenedione and DHEA all 20 samplings that were nonselective for those two steroids were also nonselective for cortisol (Figure 3B,C). For those two steroids, unsuccessful sampling involved the right adrenal vein in 19 procedures and the left in one procedure, with again no procedures unsuccessful for both adrenal veins.

Increasing the cut‐off to ≥ 3 for 11‐deoxycortisol, androstenedione and DHEA minimally reduced numbers of successful samplings. There were 15 procedures that were nonselective for cortisol at a selectivity index cut‐off of ≥ 2 that were selective for 11‐deoxycortsol at the higher cut‐off of ≥ 3 (Figure 3A). For those 15 procedures that were bilaterally selective for 11‐deoxycortsiol but not cortisol, 13 and 12 were respectively bilaterally successful for androstenedione and DHEA at the cut‐off of ≥ 3 (Figure 3BC). At a cut‐off of ≥ 3 for DHEA and ≥ 2 for cortisol, there was one procedure that was selective for cortisol and nonselective for DHEA (Figure 3C). This was, however, the exception.

### 3.4 Bilateral Rescue

At a selectivity index cut‐off of ≥ 3, proportions of bilaterally selective AVS procedures ranged from 89% to 91% for DHEA, androstenedione and 11‐deoxycortisol, all considerably higher (*p* < 0.0001) than the 69% for cortisol (Table 1). Among the 72 samplings that appeared bilaterally unsuccessful for cortisol at the cut‐off of ≥ 3, 65%–72% could be rescued with use of DHEA, androstenedione and 11‐deoxycortisol to assess selectivity. Furthermore, 26 patients showed biochemical cure after subsequent adrenalectomy, while one other showed lack of cure.

**TABLE 1:** Bilaterally successful AVS procedures according to selectivity cut‐offs ≥ 2 or ≥ 3 for the four investigated steroids.

Steroid	Cut‐off ≥ 3		Cut ≥ 2	
Cortisol	68.6%	(157/229)	84.7%	(194/229)
11‐Deoxycortisol	91.3%	(209/229)	93.0%	(213/229)
Androstenedione	90.4%	(207/229)	91.3%	(209/229)
DHEA	89.1%	(204/229)	91.3%	(209/229)

The proportion of bilaterally successful procedures for cortisol increased considerably from 69% to 85% at the lower cut‐off of ≥ 2, but this remained lower (*p* < 0.0001) than the success rates of 91%–93% for the three other steroids. Among the 35 samplings that were bilaterally unsuccessful for cortisol at the cut‐off of ≥ 2, 54% could be rescued with use of 11‐deoxycortisol to assess selectivity, while 43% could be rescued with use of either androstenedione or DHEA. Thirteen patients who subsequently underwent adrenalectomy showed biochemical cure at outcome assessment.

Even at a selectivity index cut‐off of ≥ 3 for each of those other steroids, bilateral success was higher for 11‐deoxycortisol (*p* < 0.0001), androstenedione (*p* = 0.0003) and DHEA (*p* = 0.0073) than for cortisol at the cut‐off of ≥ 2. At those different cut‐offs, rescue of unsuccessful samplings for cortisol was possible with 11‐deoxycortisol for 43% of procedures.

### 3.5 Selectivity Index Cut‐Offs

Use of ROC tables to compare diagnostic performance of each steroid for selectivity at different cut‐offs indicated optimal cut‐offs of 1.81 for cortisol, 2.68 for 11‐deoxycortisol, 2.35 for androstenedione and 2.46 for DHEA (Supporting Information S1: Table 1). At those cut‐offs specificity was consistently 100% for each steroid, while sensitivity varied from 97.5% for cortisol to between 99.8% and 100% for the other three steroids.

### 3.6 AVS Lateralisation

**FIGURE 4:**
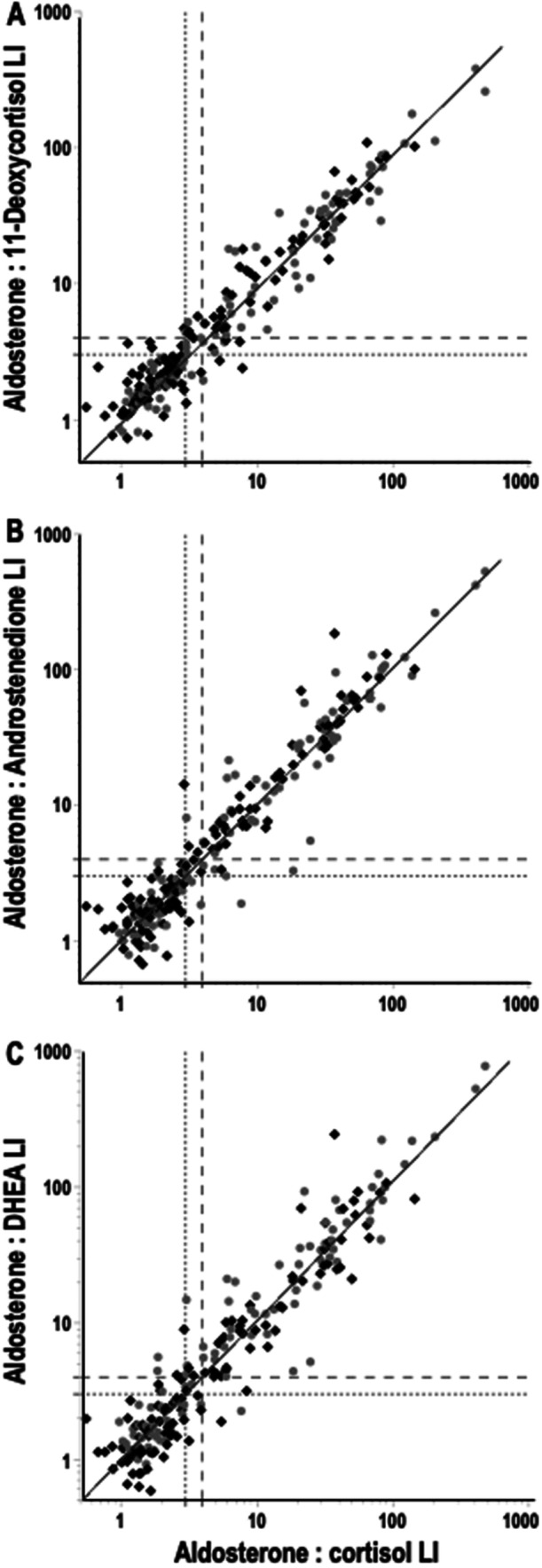
Scatterplot relationships for lateralisation indices of aldosterone relative to cortisol shown in x‐axes versus aldosterone to 11‐deoxycortisol (A), androstenedione (B) and DHEA (C) shown in y‐axes. Note the logarithmic scales for both x‐ and y‐axes. Lateralisation index values are shown separately for lateralisation larger than 1 to the right (♦) and larger than 1 at the left (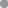). Lateralisation index cut‐offs for 3 (dotted lines) and 4 (dashed lines) are shown to demarcate lateralisation index values below which sampling may be considered non‐lateralized and above which sampling may be considered to be lateralized.

For the calculation of lateralisation indices, AVS procedures were excluded if selectivity at either adrenal vein did not meet a threshold of ≥ 2 for the three alternative steroids to cortisol. Lateralisation indices were then determined using aldosterone as the numerator and each of the four investigated steroids (cortisol, 11‐deoxycortisol, androstenedione, and DHEA) as denominators. To facilitate the display of data according to left versus right lateralisation in the same plots, lateralisation indices were averaged to establish lateralisation that tended to the right versus the left (i.e., > 1 vs. < 1). This distinction was then applied to establish AVS procedures with lateralisation indices that fell above or below cut‐offs of 3 and 4 according to overall tendency for right and left lateralisation (Figure 4).

Scatterplot relationships of lateralisation indices of aldosterone to cortisol as the routine standard (x‐axes) versus those for aldosterone to each of the three other steroids all showed close alignment with lines of identity (Figure 4). Although lateralisation indices for aldosterone to 11‐deoxycortisol showed a tendency to 14% lower values than those of aldosterone to cortisol (Figure 4A), this did not reach significance (*p* = 0.1799). In contrast, lateralisation indices of aldosterone to androstenedione were 12% higher (*p* = 0.0133) than those for aldosterone to cortisol (Figure 4B), and those of aldosterone to DHEA were 26% higher (*p* = 0.0066) than for aldosterone to cortisol (Figure 4C).

As derived from the data in Figure 3 and displayed in Table 2, there was general concordance of lateralisation indices above and below cut‐offs of 3 or 4 for lateralisation assessed using cortisol as the denominator term with those derived using 11‐deoxycortisol, androstenedione and DHEA as denominator terms. Agreement ranged from 93% to 95% at a lateralisation index cut‐off of 4 and 90% to 94% at a cut‐off of 3.

**TABLE 2:** Concordance of lateralisation indices of aldosterone to cortisol with lateralisation indices of aldosterone to 11‐deoxycortisol, androstenedione and DHEA.

	Aldosterone: 11‐Deoxycortisol		Aldosterone: Androstenedione		Aldosterone: DHEA	
LI < 3	93.8%	(90/96)	92.7%	(89/96)	87.5%	(84/96)
LI ≥ 3	94.9%	(111/117)	94.0%	(110/117)	91.5%	(111/117)
Combined^a^	94.4%	(201/213)	93.4%	(199/213)	89.7%	(191/213)
LI < 4	94.4%	(102/108)	96.3%	(104/108)	90.7%	(98/108)
LI ≥ 4	91.4%	(96/105)	94.3%	(99/105)	97.1%	(102/105)
Combined^a^	93.0%	(198/213)	95.3%	(203/213)	93.9%	(200/213)

*Note*: Concordances were calculated as proportions of results for which there was agreement of lateralisation indices (LI) below (<) and equal to or above (≥) lateralisation indices of 3 and 4 for aldosterone to cortisol versus aldosterone to 11‐deoxycortisol, androstenedione and DHEA.

^a^ Date are also shown for combined agreement.

## 4 Discussion

This study demonstrates that use of 11‐deoxycortisol, androstenedione and DHEA as alternatives to cortisol significantly improves assessments of catheter positioning to enable rescue of AVS procedures that otherwise would be deemed as failed. The findings add to an enlarging body of evidence that indicates cortisol is not an ideal biomarker to assess selectivity of AVS [15, 16, 26, 27, 28, 29, 30, 31, 32, 33]. Inferiority of cortisol compared to other biomarkers of AVS selectivity is, however, more relevant for unstimulated than cosyntropin‐stimulated sampling. For stimulated sampling, rates of successful catheterisation are relatively high and may not differ compared to assessments using other steroid biomarkers such as androstenedione and DHEA [34]. Nevertheless, given that cosyntropin‐stimulated sampling appears to offer no advantage for patient outcomes compared to unstimulated sampling [21], and may also lead to missed identification of lateralized aldosterone secretion [19, 20, 35], any advantage of improved selectivity becomes questionable with availability of superior biomarkers to make that assessment.

Among alternative biomarkers to cortisol for assessment of AVS selectivity, most studies have focused on the metabolite of epinephrine, metanephrine [16, 27, 30, 31, 32, 33], which follows from our own observations in 2013 [15]. Measurements of metanephrine not only provide a superior method over cortisol to assess AVS selectivity, but may also offer an advantage to assess lateralisation in patients with asymmetric cortisol secretion or co‐secretion of cortisol with aldosterone [16, 36, 37]. Mass spectrometry‐based measurements of metanephrine are now also widely available for screening of catecholamine‐producing tumours, which provides increased opportunities for these measurements as an alternative to cortisol.

Compared to metanephrine, studies that have examined alternative steroids to cortisol for use in AVS have been limited and of an insufficient sample size to firmly establish any statistical superiority to cortisol other than establishing higher values for selectivity indices [26, 27, 28]. One study that found no added utility was limited to cosyntropin‐stimulated sampling [34]. Steroids examined included 11‐deoxycortisol [26], 17‐hydroxyprogesterone [27] and the androgens ‐ androstenedione and DHEA [27, 28, 34]. We focused on three of those steroids, rather than metanephrine, based on analytical practicalities of mass spectrometry for measurements of multiple steroids in a single plasma specimen. For the increasing number of laboratories using this technology, such measurements offer considerable advantages in analytical accuracy over immunoassay measurements of steroids. Apart from improved freedom from analytical interferences, simultaneous measurements of multiple steroids in a single plasma sample have value not only to assist subtyping of patients with PA, but also to evaluate other disorders of adrenal steroidogenesis.

For those laboratories that for now remain restricted to immunoassays for steroids, it will be preferable where available to employ measurements of metanephrine by mass spectrometry in place of immunoassays of cortisol or other steroids. While simultaneous mass spectrometric measurements of metanephrines with steroids would be highly desirable, this is made largely impractical by the differences in chemistry of the highly polar amines and non‐polar steroids.

For centres with access to mass spectrometric measurements of aldosterone and other steroids, the present study demonstrates that substituting cortisol with 11‐deoxycortisol, androstenedione or DHEA can enable rescue of close to 50% or more of AVS procedures that would otherwise be deemed unsuccessful and have to be repeated. Moreover, these alternative steroids performed similarly to use of cortisol as a denominator normaliser to distinguish lateralized from non‐lateralized aldosterone secretion.

Apart from the aforementioned findings, the present study adds support to a previous consensus statement of opinion about appropriate selectivity index cut‐offs for unstimulated and cosyntropin‐stimulated sampling [8]. In particular, a selectivity index for cortisol close to 2 rather than 3 appears optimal to minimise numbers of nonselective samplings with non‐stimulated sampling. Nevertheless, the aforementioned cut‐offs remain arbitrary. Use of ROC curve analyses suggested that a cut‐off of 1.81 was optimal for cortisol, whereas cut‐offs of between 2.44 and 2.68 were optimal for use of 11‐deoxycortisol, androstenedione and DHEA.

Finally, although comparisons of lateralisation indices for aldosterone relative to cortisol and other steroids showed good agreement for over 90% of samplings, those data also clarify that around the commonly employed lateralisation index cut‐off of 3 there can be between 6% to 10% of samplings that are discordantly non‐lateralized by some measures and lateralized by others. Thus, as indicated by others [38, 39, 40], lateralized aldosterone secretion may not always establish unilateral PA, while conversely non‐lateralized aldosterone secretion does not always indicate bilateral disease. As shown in the present study, the discordance appears more acute at lateralisation indices below 10. Although expected, discordance at lower rather than higher laterization indices presumably accounts for findings that among patients without PA, AVS can indicate mild lateralisation in close to a fifth of procedures [23]. This can lead to unnecessary adrenalectomy. Thus, before any patient with suspected PA is committed to AVS, the diagnosis should be firmly established, usually best achieved by a confirmatory test that employs mass spectrometric measurements of aldosterone.

The study has some limitations but also strengths. A strength of this study is its relatively high sample size and multicenter nature. A limitation resides in the cross‐sectional nature of the presented analyses with outcome measures that were largely restricted to bilaterally successful catheterisation and concordance of lateralisation. Nevertheless, the cohort design of the PROSALDO trial did allow for some assessment of post‐adrenalectomy outcomes. The 37% of patients with failed selectivity by cortisol who showed biochemical cure after adrenalectomy indicates that in addition to more conclusive confirmation of correct positioning of catheters, the three alternative steroids to cortisol may also increase proportions of patients who undergo curative adrenalectomy. This conclusion should, however, be tempered by the study design, which did not expressly stipulate that results for cortisol at AVS should be over‐ridden by results of the other steroids to guide subsequent decision‐making. A randomised controlled trial in which outcomes after use alternative steroids are compared to outcomes after cortisol would better provide evidence for improved patient outcomes.

As further detailed in the supplement, there is considerable variability in how AVS is practiced among different centres. The present study clarifies use of alternative steroids to cortisol only for unstimulated AVS. Moreover, use of such alternative steroids is best confined to centres that employ mass spectrometry. This is because measurements by mass spectrometry are considerably more accurate than by immunoassay and also carry the advantage of enabling measurements of multiple steroids in a single analysed specimen (multiplexing). Although sample throughput is lower and instrumentation is more costly and requires more highly skilled operators compared to immunoassay platforms, the advantages of high accuracy and multiplexing are considerable. For steroid panels this goes well beyond AVS to diagnostic stratification of many disorders of steroidogenesis, all under a single analytical method.

## Supporting information

AVS‐Selectivity‐Supplement110825.

## Data Availability

The data that support the findings of this study are available from the corresponding author upon reasonable request.
