# Supplementary material for: Mass Spectrometric Measurements of 11‐Deoxycortisol, Androstenedione and Dehydroepiandrosterone Are Superior to Cortisol to Assess Selectivity of Non‐Stimulated Adrenal Vein Sampling
Source: Clin Endocrinol (Oxf). 2025 Sep 16;104(1):10–8. doi: 10.1111/cen.70037 (PMC12669813; doi:10.1111/cen.70037)
Supplement: Supplementary file 1 — AVS‐Selectivity‐Supplement110825. [file CEN-104-10-s001.docx]

**Mass spectrometric measurements of 11-deoxycortisol, androstenedione and dehydroepiandrosterone are superior to cortisol to assess selectivity of non-stimulated adrenal vein sampling**

Francesco Alessi^1^, Christina Pamporaki^1^, Mirko Peitzsch^2^, Georgiana Constantinescu^1^, Hanna Remde^3^, Lydia Kürzinger^3^, Carmina T Fuss^3^, Manuel Schulze^4^, Sybille Fuld^1^, Sradha Kotwal^5^, Jun Yang^6^, Martin Reincke^7^, Felix Beuschlein^7,8^, Jacques W.M. Lenders^9^ and Graeme Eisenhofer^1^

^1^Department of Medicine III, University Hospital Carl Gustav Carus, Technische Universität Dresden, Germany; ^2^Institute of Clinical Chemistry and Laboratory Medicine, Medical Faculty and University Hospital Carl Gustav Carus, Technische Universität Dresden, Germany; ^3^Department of Internal Medicine I, Division of Endocrinology and Diabetes, University Hospital, University of Würzburg, Germany; ^4^Center for Interdisciplinary Digital Sciences, Department Information Services and High Performance Computing, Technische Universität Dresden, Germany; ^5^Department of Renal Medicine, St George Hospital, UNSW Medicine and Health, Sydney, Australia; ^6^Centre for Endocrinology and Metabolism, Hudson Institute of Medical Research, Clayton, Australia; ^7^Department of Medicine IV, University Hospital, Ludwig Maximilian University Munich, Munich; ^8^Department of Endocrinology, Diabetology and Clinical Nutrition, University Hospital Zurich and the LOOP Zurich Medical Research Center, Zurich, Switzerland; ^9^Department of Internal Medicine, Radboud University Medical Center, Nijmegen, the Netherlands.

**Contents**

**___________________________________________________________________________**

Section Page

Study design and patient flow…………………………………………………………….…………... 2

Supplemental figure 1…………………………………………………………………….………..…. 2

Receiver-operating characteristic curve analyses……………………………………….……………. 4

Supplemental table 1.………………………………………………………………….…………...…. 4

Inter-center variability in clinical practice procedures………………………………………….…….. 5

References.………………………………………………………………….……………………...…. 6

**___________________________________________________________________________**

This supplemental appendix is primarily provided to address comments from reviewers that required additional illustrative material and text that were outside of journal guidelines for maximum numbers of figures/tables and word length requirements. Requests by reviewers included incorporation of a flow a diagram, analyses of receiver-operating characteristic curves and discussion of inter-center variability in clinical practice procedures.

**Study design and patient flow**

The prospective study on the diagnostic value of steroid profiling in primary aldosteronism (PROSALDO) is a registered international multicenter trial (trial registration no: DRKS00017084 <https://drks.de/search/en/trial/DRKS00017084>) designed for a primary objective to evaluate the combination of mass spectrometry-based steroid profiling and machine learning for improved diagnostic stratification of patients with suspected primary aldosteronism. Recruitment of patients began in January 2019 and remains ongoing until all study objectives are satisfied. Nevertheless, recruitment requirements for the primary objective were satisfied at the end of 2023, but required outcome assessments that were largely completed within the following year. One of several secondary objectives of the study was to determine the value of mass spectrometric measurements of adrenal-derived steroids for adrenal venous sampling (AVS) based subtyping of patients with primary aldosteronism. The presently reported sub-study represents a cross-sectional evaluation of three selected steroids (11-deoxycortisol, androstenedione and dehydroepiandrosterone) as alternatives to cortisol to determine selective catheterization of adrenal veins during AVS (Supplemental figure 1).

Details about the protocol and the flow of patients through the prospective cohort study have been described in several earlier manuscripts: 1. two first reports that described severe macromolecular interferences with immunoassay measurements of aldosterone that largely invalidates use of those assays when used for disease confirmation [1, 2]; 2. a report that described integration of steroid-profiling based machine learning models within a clinical decision support system [3]; 3. a report that focused on diagnostic performance of the aldosterone to renin ratio (ARR) for screening [4]; 4. a report that identified interfering influences of antihypertensive drugs on the ARR, renin, aldosterone, hybrid steroids and outputs of machine learning-based models for screening [5]; 5. a report that validated use of mass spectrometric measurements of aldosterone during the seated saline infusion test for confirmation of primary aldosteronism [6]. Details of the PROSALDO trial are covered in the aforementioned publications and associated supplements. Those details are again repeated here as follows.

**Supplemental figure 1.** Patient flow though the PROSALDO trial from initial recruitment to final outcome assessment. The present report focuses on a single phase (AVS subtyping) in the flow of patients through the protocol. Therefore, the study involves a cross-sectional design. However, the study also reports on some patients who underwent AVS and reached outcome assessment, so also retains an element of the overall cohort design.

As illustrated in supplemental figure 1, the general flow of patients through the study followed recommendations of the 2016 Endocrine Society guideline [7]. Initial screening employed the ARR, which required two or more measurements of aldosterone and renin on separate days. At least one measurement had to be performed with patients not taking antihypertensive medications known to interfere with measurements of renin and aldosterone. If required, antihypertensive therapy was adjusted to an alpha-adrenoceptor blocker and/or a non-dihydropyridine calcium channel blocker without known impacts on the renin-angiotensin-aldosterone system. Isolated exceptions included patients in whom adjustments to medications were not possible and in whom directional differences in the ARR relative to known impacts of interfering drugs were not compatible with established influences to cause false-positive result (i.e., a normal ARR relative to upper cut-offs in a patient exclusively taking a beta-adrenoceptor blocker) or a false-negative result (i.e., elevations in the ARR relative to upper cut-offs in patients taking antihypertensive agents known to elevate plasma concentrations of renin and lower the ARR). Renin was measured as direct renin concentrations by either of two chemiluminescence immunoassays from either DiaSorin (Liaison) Immuno Diagnostic Systems (iSYS). Aldosterone was measured at three centers by the DiaSorin Liaison chemiluminescence immunoassay, at two centers by the Immuno Diagnostic Systems iSYS chemiluminescence immunoassay and for two centers by mass spectrometry.

In addition to the routine measurements of aldosterone and renin, samples of plasma were also collected at each phase of the protocol for mass spectrometric measurements of aldosterone and other steroids. Those samples were shipped to the Dresden laboratory where mass spectrometric measurements were performed and data were then uploaded into electronic case report forms (eCRFs), as supported by a research electronic data capture (REDCap) tool [8, 9]. In addition to collection of all required data for the PROSALDO protocol, the REDCap system also facilitated automated generation of reports that included steroid profiles, associated reference intervals, and machine learning-based interpretations of steroid profiles. Following expert review, investigators at study centers were provided with reports as PDF files. Thus, as illustrated in supplemental figure 1, the protocol involved use of steroid profile derived machine learning-probability scores as a separate tool from the ARR for paired comparisons of the ARR and machine learning probability scores for screening. Non-suppressed plasma renin and normal ARRs have been described previously among patients with unilateral primary aldosteronism in whom cure was established after resection of diseased adrenals [10]. This feature of the PROSALDO trial enabled identification of several similarly characterized patients who had non-suppressed renin and surgically curable unilateral primary aldosteronism.

Positive results at screening for either or both the ARR and steroid profile-derived probability scores were followed by the seated saline infusion test (Supplemental figure 1), which was performed according to procedures outlined elsewhere [6]. A positive result for the seated saline infusion test was initially defined according to post-infusion plasma concentrations of aldosterone above 170 pmol/L according to measurements performed locally at each study center and mostly by immunoassay measurements. However, during the early stages of the protocol it was determined that results for immunoassay measurements of aldosterone were over-estimated compared to mass spectrometric measurements [1, 2]. The over-estimation was more commonly observed and showed larger extents of overestimation with use of the Liaison than the iSYS immunoassay. Interferences were also patient specific, highly variable and proportionally more extensive at lower concentrations such as during suppression of plasma aldosterone Subsequently, interpretation of test results was based on measurements of aldosterone by mass spectrometry at Dresden and the two centers in Sydney for which measurements were carried out in a single laboratory at the Prince of Wales Hospital. The cut-off for a positive test result was set at ≥162 pmol/L, as defined by Thuzar and colleagues in 2020 for mass spectrometric measurements of aldosterone [11]. The seated saline infusion test was further validated for mass spectrometric measurements of aldosterone in 420 patients of the PROSALDO trial [6]. An optimal cut-off was defined at 169 pmol/L, which provided sufficient sensitivity and specificity (96% each) for disease confirmation and exclusion provided that standard operating procedures were appropriately followed.

Progression of patients to AVS was stipulated under the protocol to require positive results for one or both screening tests and a positive result for the seated saline infusion test (Supplemental figure 1). However, occasional patients progressed directly to AVS from screening in cases where positive screening test results were unlikely to be false-positive and where the presentation was strongly consistent with a diagnosis of primary aldosteronism. Also, in the early stages of the protocol, before immunoassay inaccuracy was recognised, progression to AVS was based on immunoassay measurements of aldosterone. This persisted for some centers where there was perceived need for continued reliance on certified immunoassays. Progression to AVS also required willingness of patients to undergo adrenalectomy according to results of subtyping, including imaging studies.

The PROSALDO protocol stipulated that all patients undergo follow-up outcome assessments at six months or more after the last test or intervention (Supplemental figure 1). Follow-up of patients who underwent adrenalectomy was according to the Primary Aldosteronism Surgical Outcome (PASO) criteria for assessments of biochemical or clinical cure [12]. Additionally, immunohistochemical staining was carried out according to international consensus criteria (HISTALDO) to confirm that excised adrenal contained aldosterone synthase positive lesions [13].

**Receiver-operating characteristic curve analyses**

Receiver-operating characteristic (ROC) curves to examine optimal cut-offs of selectivity indices for each of the four steroids were constructed using combined results of selectivity indices for right and left adrenal vein samplings from the 229 patients (n=458). For this it was necessary to establish a classification system to define selective sampling. Since use of any single measure would introduce confirmation or reference bias to favour that measure over all others, we chose to use a combination of all four measures. Specifically, selective sampling was defined according to any single sampling that was characterized by at least three of the four steroid measures with selectivity indices ≥2. From the subsequent analyses of ROC curves for each steroid measure ROC tables were generated, selected results of which are displayed in supplementary table 1.

| **Supplementary table 1.** Receiver operating characteristic curve table for analysis of diagnostic performance of each of the four steroids at different selectivity index cut-offs, including the optimal cut-off as assessed from Youden's index* | | | | | | | |
| --- | --- | --- | --- | --- | --- | --- | --- |
|  | Selectivity index | Sensitivity | Specificity | TP | TN | FP | FN |
| Cortisol | | |  |  |  |  |  |
|  | 3.00 | 86.8% | 100.0% | 380 | 20 | 0 | 58 |
|  | 2.00 | 95.4% | 100.0% | 418 | 20 | 0 | 20 |
| * | 1.81 | 97.5% | 100.0% | 427 | 20 | 0 | 11 |
|  | 1.58 | 98.9% | 90.0% | 433 | 18 | 2 | 5 |
|  | 1.21 | 100.0% | 80.0% | 438 | 16 | 4 | 0 |
| 11-Deoxycortisol | | |  |  |  |  |  |
|  | 3.00 | 99.5% | 100.0% | 436 | 20 | 0 | 2 |
| * | 2.68 | 99.8% | 100.0% | 437 | 20 | 0 | 1 |
|  | 2.56 | 100.0% | 95.0% | 438 | 19 | 1 | 0 |
| Androstenedione | | |  |  |  |  |  |
|  | 3.00 | 99.8% | 100.0% | 437 | 20 | 0 | 1 |
|  | 2.44 | 99.8% | 100.0% | 437 | 20 | 0 | 1 |
| * | 2.35 | 100.0% | 100.0% | 438 | 20 | 0 | 0 |
| Dehydroepiandrosterone | | |  |  |  |  |  |
|  | 3.00 | 99.1% | 100.0% | 434 | 20 | 0 | 4 |
| * | 2.46 | 99.8% | 100.0% | 437 | 20 | 0 | 1 |
|  | 2.00 | 100.0% | 100.0% | 438 | 19 | 1 | 0 |
| Abbreviations: TP, true positives; TN, true negatives; FP, false positives; FN, false negatives | | | | | | | |

Results of the ROC curve analyses suggested an optimal selectivity index cut-off for cortisol of 1.81 that achieved 100% specificity at a sensitivity of 97.5% (Supplementary figure 1). The improved sensitivity was associated with decreased numbers of false-negative results from 58 and 20 at respective selectivity indices of ≥3 and ≥2 to 11 at the selectivity index of 1.81. Optimal selectivity indices for 11-deoxycortisol, androstenedione and dehydroepiandrosterone were 2.68, 2.35 and 2.46 respectively, which all offered 100% specificity for assured selective sampling at sensitivities of at least 99.8% (false negatives ≤1). The limitation of this analysis is that the classification system for defining selective versus non-selective sampling (three of four steroids with selectivity indices ≥2) was based on an existing arbitrary cut-off for selectivity indices (selectivity indices ≥2).

**Inter-center variability in clinical practice procedures**

In addition to considerable inter-center variability in methods to screen for primary aldosteronism and subsequent use of confirmatory tests, there is also considerable inter-center variability in use of AVS for subtype classification. In 2017, John Funder likened the management of primary aldosteronism to the cottage industries of the eighteenth century pre-industrial revolution era [14]. As predicted by Funder, harmonization in diagnostic and measurement procedures has not been achieved over the ensuing years due to the wide variations in clinical practice. The 2025 Endocrine Society guideline does little to address the “cottage industry” problem and how to bring about a more evidence-based harmonised approach to diagnosis and management of primary aldosteronism [15]. The outlined 10 recommendations were all conditional (lowest grade strength) and all at very low or low levels of confidence in surveyed evidence. This clarifies limited progress in diagnosis and management since the 2016 guideline and leaves clinicians with little clarity to move forward with solutions to this common but largely undiagnosed cause of hypertension.

Variability in procedures employed for AVS, as mentioned in the main text, include use of cosyntropin-stimulated versus non-stimulated sampling as well as at some centers non-stimulated sampling followed by stimulated sampling. As demonstrated in several studies that reported on both non-stimulated and stimulated sampling, results for selectivity and lateralization can vary considerably during the two sampling procedures [16-21]. It was, however, consistently established that selective sampling according to measurements of cortisol are more conclusively established with than without cosyntropin stimulation. On the other hand, considerable discordance in lateralization has been observed with a general shift from lateralized to non-lateralized aldosterone secretion with stimulated sampling; in some procedures even complete discordance between right and left lateralization has been observed for stimulated versus stimulated samplings [17]. In the largest of those retrospective studies, which involved 1625 AVS procedures, no differences in outcomes in terms of cured unilateral cases were reported for non-stimulated versus stimulated procedures [20]. It was also concluded that commonly used cut-offs for selectivity and lateralization were associated with disappointingly low rates of successful catheterization and identified unilateral disease. Lack of impact of non-stimulated versus stimulated sampling on outcomes was subsequently more clearly defined in a randomized controlled study [22]. Another more recent retrospective study also failed to establish any difference in post-surgical outcomes with stimulated versus non-stimulated sampling [23]. Nevertheless, it is argued by some investigators that findings of consistent lateralization with both non-stimulated and stimulated sampling can better inform decisions about adrenalectomy [24].

Stimulated sampling may be achieved by either a bolus injection or continuous infusion of cosyntropin [16]. Sampling is most usually carried out with a single catheter placed first in one adrenal vein (usually the more difficult to access right adrenal vein) and then the other (usually the left) or alternatively may be carried out simultaneously using two catheters. Other variations in procedures include the timing of sampling, usually stipulated at 15 minutes after the start of the cosyntropin infusion; however, early sampling time points of 5 to 10 minutes after the start of the infusion do not appear to consistently impact measures of selectivity or lateralization; however, for a single reported patient among 46 there was discordance in lateralization between an earlier and a later sampling time point that was concordant with non-stimulated sampling [25].

Non-stimulated sampling used alone, although less complicated than stimulated sampling, nevertheless is similarly technically difficult and requires the skills of experienced interventional radiologists. As in stimulated sampling, non-stimulated AVS may be carried with simultaneous or sequential catheterization of adrenal veins, though beyond this there are lesser procedural variations compared to stimulated sampling. The more technically demanding simultaneous sampling procedure has the potential advantage to avoid impacts of pulsatile aldosterone and cortisol secretion, which may compromise interpretation of lateralized versus non-lateralized aldosterone secretion with sequential sampling [26]. For all sampling practices, there is also need to consider how patients should be prepared for AVS and whether the procedure should be confined to a particular time of the day or strictly performed on an in-patient basis. There has been previous recommendations reached by consensus that hypokalemia should be corrected and antihypertensive medications adjusted before AVS, which should be conducted in the morning hours for non-stimulated sampling [27]. These recommendations were, however, based on expert opinion rather than evidence and have undergone some revision in a more recent review of AVS [28] as well as in the 2025 Endocrine Society guideline [15]. In particular, some earlier recommendations have been found to be impractical to follow for many patients and procedures [29, 30]. It is now recommended that blood pressure should be well controlled during AVS and as that as long as renin remains suppressed there is no need to withdraw or adjust antihypertensive medications [15]. The need to conduct AVS during morning hours has also been challenged by a study that showed no differences in assessments of selectivity and subtype diagnosis for AVS performed in afternoon versus morning hours [31].

The 2025 Endocrine Society guideline recognises all aforementioned stimulated and non-stimulated procedures, including those with simultaneous or sequential sampling [15]. Relative benefits are covered, as also outlined above, and no procedure is recognised to provide any overall benefit over the others except for the improved assessments of selectivity for stimulated versus non-stimulated sampling procedures. Technical difficulties are rightfully recognised together with the high cost and some potential risks of AVS. No mention is made of alternative biomarkers to cortisol to assess selectivity; this includes measurements of plasma metanephrine, this despite the already clear evidence about the advantages of this biomarker over cortisol as outlined in over seven independent original reports [32-38]. It will likely take many more reports, ideally including randomised controlled trials, before the shortcomings of cortisol are recognised and some degree of harmonisation in variable center-to-center procedures is reached. Better still, AVS will be replaced by functional imaging. In the interim, AVS remains a part of the cottage industries of clinical practices for diagnosis and management of primary aldosteronism.

**References**

1. Constantinescu G, Bidlingmaier M, Gruber M, et al. Mass spectrometry reveals misdiagnosis of primary aldosteronism with scheduling for adrenalectomy due to immunoassay interference. Clin Chim Acta. 2020;507:98-103.

2. Eisenhofer G, Kurlbaum M, Peitzsch M, et al. The Saline Infusion Test for Primary Aldosteronism: Implications of Immunoassay Inaccuracy. J Clin Endocrinol Metab. 2022;107(5):e2027-e2036.

3. Constantinescu G, Schulze M, Peitzsch M, et al. Integration of artificial intelligence and plasma steroidomics with laboratory information management systems: application to primary aldosteronism. Clin Chem Lab Med. 2022;60:1929-1937.

4. Fuld S, Constantinescu G, Pamporaki C, et al. Screening for Primary Aldosteronism by Mass Spectrometry Versus Immunoassay Measurements of Aldosterone: A Prospective Within-Patient Study. J Appl Lab Med. 2024;9(4):752-766.

5. Constantinescu G, Gruber S, Fuld S, et al. Steroidomics-Based Screening for Primary Aldosteronism: Impact of antihypertensive Drugs. Hypertension. 2024;81(10):2060-2071.

6. Pamporaki C, Remde H, Constantinescu G, et al. The saline infusion test with mass spectrometric measurements of aldosterone to confirm primary aldosteronism. J Hypertens. 2025;“in press”.

7. Funder JW, Carey RM, Mantero F, et al. The management of primary aldosteronism: case detection, diagnosis, and treatment: An Endocrine Society Clinical Practice Guideline. J Clin Endocrinol Metab. 2016;101:1889-1916.

8. Harris PA, Taylor R, Thielke R, et al. Research electronic data capture (REDCap)--a metadata-driven methodology and workflow process for providing translational research informatics support. J Biomed Inform. 2009;42(2):377-381.

9. Harris PA, Taylor R, Minor BL, et al. The REDCap consortium: Building an international community of software platform partners. J Biomed Inform. 2019;95:103208.

10. Jansen PM, Stowasser M. Aldosterone-producing adenoma associated with non-suppressed renin: a case series. J Hum Hypertens. 2022;36(4):373-380.

11. Thuzar M, Young K, Ahmed AH, et al. Diagnosis of Primary Aldosteronism by Seated Saline Suppression Test-Variability Between Immunoassay and HPLC-MS/MS. J Clin Endocrinol Metab. 2020;105(3):e477-e483.

12. Williams TA, Lenders JWM, Mulatero P, et al. Outcomes after adrenalectomy for unilateral primary aldosteronism: an international consensus on outcome measures and analysis of remission rates in an international cohort. Lancet Diabetes Endocrinol. 2017;5(9):689-699.

13. Williams TA, Gomez-Sanchez CE, Rainey WE, et al. International Histopathology Consensus for Unilateral Primary Aldosteronism. J Clin Endocrinol Metab. 2021;106(1):42-54.

14. Funder JW. Primary Aldosteronism: The Next Five Years. Horm Metab Res. 2017;49(12):977-983.

15. Adler GK, Stowasser M, Correa RR, et al. Primary Aldosteronism: An Endocrine Society Clinical Practice Guideline. J Clin Endocrinol Metab. 2025.

16. Monticone S, Satoh F, Giacchetti G, et al. Effect of adrenocorticotropic hormone stimulation during adrenal vein sampling in primary aldosteronism. Hypertension. 2012;59(4):840-846.

17. El Ghorayeb N, Mazzuco TL, Bourdeau I, et al. Basal and Post-ACTH Aldosterone and Its Ratios Are Useful During Adrenal Vein Sampling in Primary Aldosteronism. J Clin Endocrinol Metab. 2016;101(4):1826-1835.

18. Yatabe M, Bokuda K, Yamashita K, et al. Cosyntropin stimulation in adrenal vein sampling improves the judgment of successful adrenal vein catheterization and outcome prediction for primary aldosteronism. Hypertens Res. 2020;43(10):1105-1112.

19. Sung TY, Alobuia WM, Tyagi MV, et al. Adrenal Vein Sampling to Distinguish Between Unilateral and Bilateral Primary Hyperaldosteronism: To ACTH Stimulate or Not? J Clin Med. 2020;9(5).

20. Rossitto G, Amar L, Azizi M, et al. Subtyping of Primary Aldosteronism in the AVIS-2 Study: Assessment of Selectivity and Lateralization. J Clin Endocrinol Metab. 2020;105(6).

21. Yozamp N, Hundemer GL, Moussa M, et al. Adrenocorticotropic Hormone-Stimulated Adrenal Venous Sampling Underestimates Surgically Curable Primary Aldosteronism: A Retrospective Cohort Study and Review of Contemporary Studies. Hypertension. 2021;78(1):94-103.

22. Yang S, Du Z, Zhang X, et al. Corticotropin Stimulation in Adrenal Venous Sampling for Patients With Primary Aldosteronism: The ADOPA Randomized Clinical Trial. JAMA Netw Open. 2023;6(10):e2338209.

23. Kobayashi H, Nakamura Y, Abe M, et al. Assessing Lateralization Index of Adrenal Venous Sampling for Surgical Indication in Primary Aldosteronism. J Clin Endocrinol Metab. 2025;110(4):e1084-e1093.

24. Younes N, Larose S, Bourdeau I, et al. Role of Adrenal Vein Sampling in Guiding Surgical Decision in Primary Aldosteronism. Exp Clin Endocrinol Diabetes. 2023;131(7-08):418-434.

25. Tee QX, Doery JCG, Desra A, et al. Adrenal vein sampling: accuracy of earlier sampling post adrenocorticotropic hormone (ACTH) administration. Clin Radiol. 2025;84:106861. doi: 10.1016/j.crad.2025.106861.

26. Lupi A, Battistel M, Barbiero G, et al. Simultaneous bilateral adrenal vein sampling for primary aldosteronism: useful tips to make it simple and safe. Eur Radiol. 2019;29(11):6330-6335.

27. Rossi GP, Auchus RJ, Brown M, et al. An expert consensus statement on use of adrenal vein sampling for the subtyping of primary aldosteronism. Hypertension. 2014;63(1):151-160.

28. Rossi GP, Battistel M, Seccia TM, et al. Subtyping of Primary Aldosteronism by Adrenal Venous Sampling. Endocr Rev. 2025;46(4):501-517.

29. Ching KC, Cohen DL, Fraker DL, et al. Adrenal Vein Sampling for Primary Aldosteronism: A 2-Week Protocol for Withdrawal of Renin-Stimulating Antihypertensives. Cardiovasc Intervent Radiol. 2017;40(9):1367-1371.

30. Ganesh M, Abadin SS, Fogelfeld L. Adrenal Vein Sampling Without Discontinuation of Mineralocorticoid Receptor Antagonist Therapy. Endocr Pract. 2020;26(9):953-959.

31. Yoneda M, Kometani M, Aiga K, et al. Impact of Conducting Adrenal Venous Sampling in the Morning Versus Afternoon in Primary Aldosteronism. J Endocr Soc. 2023;7(3):bvad007.

32. Dekkers T, Deinum J, Schultzekool LJ, et al. Plasma metanephrine for assessing the selectivity of adrenal venous sampling. Hypertension. 2013;62(6):1152-1157.

33. Christou F, Pivin E, Denys A, et al. Accurate Location of Catheter Tip With the Free-to-Total Metanephrine Ratio During Adrenal Vein Sampling. Front Endocrinol (Lausanne). 2022;13:842968. metanephrine ratio during adrenal vein sampling. Front Endocrinol (Lausanne). 2022;13:842968. doi: 10.3389/fendo.2022.842968

34. Buffolo F, Pieroni J, Ponzetto F, et al. Prevalence of Cortisol Cosecretion in Patients With Primary Aldosteronism: Role of Metanephrine in Adrenal Vein Sampling. J Clin Endocrinol Metab. 2023;108(9):e720-e725.

35. Kawahara EZ, Okubo J, Cavalcante A, et al. Adrenal Venous Sampling Using Metanephrine in Primary Aldosteronism With or Without Cortisol Co-Secretion. J Clin Endocrinol Metab. 2024;dgae896. doi: 10.1210/clinem/dgae896.

36. Carroll RW, Corley B, Feltham J, et al. The value of plasma metanephrine measurements during adrenal vein sampling. Endocr Connect. 2024;13(2):e230300. doi: 230310.231530/EC-230323-230300.

37. Htut Z, Alsafi A, Sharma A, et al. The Utility of Plasma Metanephrines to Optimise Adrenal Vein Sampling for Primary Aldosteronism: A Single Centre Experience. Clin Endocrinol (Oxf). 2025;doi: 10.1111/cen.15277.

38. Nadeem IM, Rahman Y, Nadeem L, et al. The Effectiveness of Metanephrine-Based Compared to Cortisol-Based Measurements to Assess Selectivity and Lateralization of Adrenal Vein Sampling in Primary Aldosteronism: A Systematic Review. J Vasc Interv Radiol. 2025;S1051-0443(25)00335-5. doi: 10.1016/j.jvir.2025.04.020.
